# Maillard Reaction in Flour Product Processing: Mechanism, Impact on Quality, and Mitigation Strategies of Harmful Products

**DOI:** 10.3390/foods14152721

**Published:** 2025-08-03

**Authors:** Yajing Qi, Wenjun Wang, Tianxiang Yang, Wangmin Ding, Bin Xu

**Affiliations:** School of Food and Biological Engineering, Jiangsu University, Zhenjiang 212013, China; xiaoyataoyao@163.com (Y.Q.); 15963608557@163.com (W.W.); 13130959645@163.com (T.Y.); 18105299720@163.com (W.D.)

**Keywords:** flour products, Maillard reaction, mitigation strategies

## Abstract

The Maillard reaction refers to the reaction between carbonyl compounds with reducing properties and amino-containing compounds that undergo condensation and polymerization to produce melanoidins. In flour product processing, the Maillard reaction is a critical chemical reaction influencing color, flavor, nutrition, and safety. A moderate Maillard reaction contributes to desirable color and flavor profiles in flour products, whereas an excessive reaction leads to amino acid loss and the formation of harmful substances, posing potential health risks. This review summarizes the substrate sources, reaction stages, influencing factors, impact on quality, and mitigation strategies of harmful products, aiming to provide a reference for regulating the Maillard reaction in flour product processing. Currently, most existing mitigation strategies focus on inhibiting harmful products, while research on the synergistic optimization of color and flavor remains insufficient. Future research should focus on elucidating the molecular mechanisms of reaction pathways, understanding multi-factor synergistic effects, and developing composite regulation technologies to balance the sensory quality and safety of flour products.

## 1. Introduction

As staple foods fundamental to global dietary cultures, flour products have evolved from solely providing energy to meeting consumers’ diverse demands for sensory quality and nutritional balance. Broadly defined, flour products can be defined as various finished or semi-finished products made mainly from flour and granular materials, with appropriate auxiliary materials added, and processed through a series of techniques such as kneading, fermentation, steaming, baking, and frying [1,2,3]. Flour and granular materials are primarily derived from crops, including Poaceae (such as wheat, maize, oat, rye, and sorghum), Fabaceae, Chenopodiaceae (such as quinoa), Polygonaceae (such as buckwheat), and Solanaceae (such as potato) [4]. Common flour products include breads, cookies, noodles, fried bread sticks, and steamed breads.

The Maillard reaction is a key reaction in flour product processing. Also known as the carbonyl–amino reaction, it refers to complex reactions in food systems where carbonyl compounds with reducing properties and amino compounds undergo condensation, rearrangement, and polymerization to form melanoidins [5,6,7]. This reaction was originally discovered by French chemist Louis Camille Maillard in 1912 during the thermal treatment of glycine and glucose mixtures [8].

A moderate Maillard reaction is key to imparting attractive sensory qualities to flour-based products. It produces the characteristic golden crust and rich baking aroma in bread, and gives cookies their unique caramel color and delightful caramel flavor. It also creates the alluring golden, crispy appearance and distinct deep-fried aroma in fried bread sticks [9]. However, an excessive Maillard reaction can trigger a series of negative effects. Visually, it manifests as excessive browning. For example, the bread crust may turn black, and cookie color can become too dark. Besides, a low-temperature Maillard reaction during storage of flour-based products with specific moisture content, such as fresh noodles, can intensify browning. Nutritionally, an excessive Maillard reaction significantly reduces the bioavailability of essential amino acids, particularly lysine. Protein digestibility decreases due to cross-linking, impairing overall nutritional value. Furthermore, high-temperature processing can produce various potentially hazardous substances, such as acrylamide (AA), 5-hydroxymethylfurfural (HMF), and advanced glycation end products (AGEs) [10,11]. AA is widely present in baked and fried flour products and has neurotoxicity, carcinogenicity, and mutagenicity [12,13]. HMF is commonly found in products such as biscuits and bread. Its metabolite, sulfoxymethylfurfural (SMF), is genotoxic and nephrotoxic [14]. When accumulated in the human body, AGEs such as Nε-carboxymethyl-lysine (CML) and Nε-carboxyethyl-lysine (CEL) can induce oxidative stress or damage nerve cells, leading to the occurrence of various diseases such as diabetes [15].

Although the Maillard reaction significantly influences flour product quality, existing reviews predominantly examine its mechanisms and effects in dairy and meat products rather than providing specialized systematic analysis for flour-based foods [16,17]. This review therefore focuses on flour products, methodically analyzing substrate sources, reaction mechanisms, influencing factors, and multidimensional mitigation strategies of the Maillard reaction during processing. The synthesis aims to establish a robust theoretical foundation and offer practical guidance for future mechanistic investigations and precision control technology development in this field.

## 2. Substrate Sources of the Maillard Reaction in Flour Product Processing

### 2.1. Carbonyl Sources

#### 2.1.1. Reducing Sugars

##### Natural Reducing Sugars in Raw Materials

There are differences in the content of natural reducing sugars among raw materials for flour products. Among barley, rye, buckwheat, oat, and wheat, the basic sequence of reducing sugar concentration is maltose exceeding glucose, which in turn exceeds fructose. Maltose levels range from 4.8 to 11.6 mg/kg, glucose from 1.5 to 4.9 mg/kg, and fructose from 0.7 to 4.5 mg/kg [18]. The reducing sugar contents across these raw materials are comprehensively detailed in Table 1.

##### Reducing Sugars from the Milling Process

**Figure 1:**
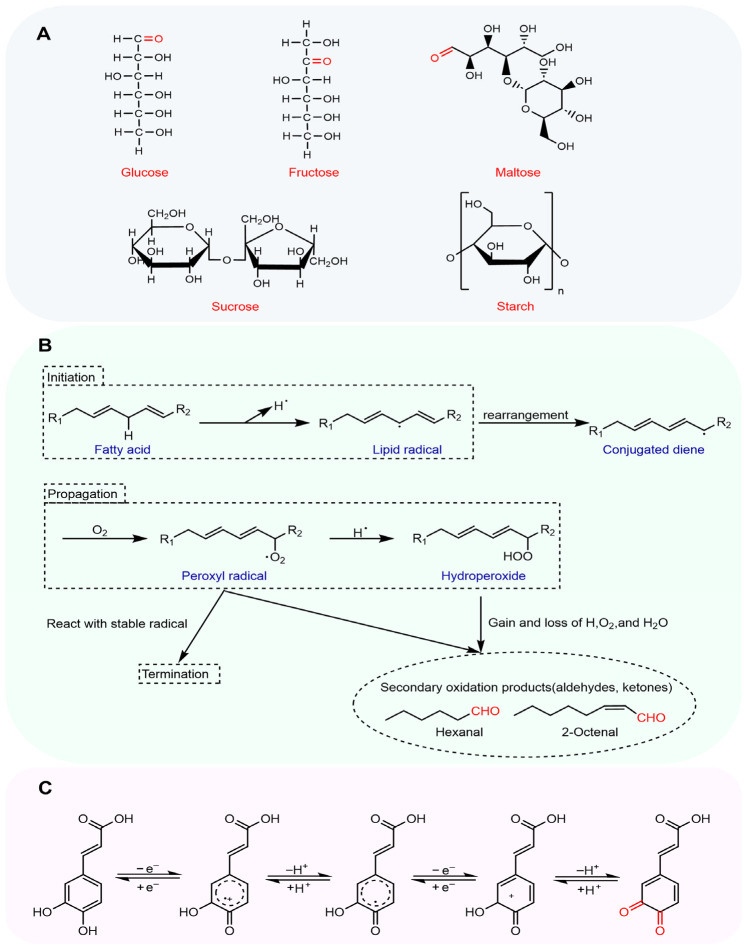
Carbonyl sources in flour product processing. (**A**) Reducing sugars; (**B**) lipid degradation pathways [44]; (**C**) o-quinone formation (using caffeic acid as an example). Note: The red color in the figure is used to highlight the carbonyl groups.

The grain kernels for flour milling contain starch and amylase. Amylase can hydrolyze starch to produce glucose and maltose. Although amylase generally has difficulty enzymatically hydrolyzing intact starch granules, it is relatively active towards damaged starch granules. During flour milling, mechanical forces such as squeezing and friction damage cellular structures and increase temperature, thereby enhancing amylase activity. Consequently, amylase decomposes the damaged starch, producing reducing sugars [20] (Figure 1A). However, due to the complexity of the milling process, there is currently a lack of targeted experimental designs to quantify the contribution of reducing sugars produced during the wheat milling process to the substrates of the Maillard reaction in various flour products. Research should be conducted on how to control the milling process based on specific flour requirements to regulate the level of reducing sugars in the flour raw materials in the future.

##### Reducing Sugars Derived from Polysaccharide Degradation During Dough Fermentation

Pure dough systems primarily involve α-amylase and β-amylase, where endogenous enzymatic activation occurs upon flour hydration [21]. α-Amylase hydrolyzes the α-1,4 glycosidic bonds in starch, converting it into dextrins [22,23]. Subsequently, β-amylase decomposes dextrins into maltose (Figure 1A), providing substrate for the Maillard reaction [24]. Yeast is sometimes added during the processing of flour products. Yeast incorporation enables metabolic production of β-glucosidase, which further hydrolyzes β-glucanase-derived low-molecular-weight oligosaccharides into reducing sugars [25]. Initial fermentation stages feature yeast invertase hydrolyzing sucrose into glucose and fructose, thus elevating reducing sugar content. However, later stages demonstrate partial accumulation of residual reducing sugars despite yeast consumption of these monosaccharides [26].

#### 2.1.2. Lipid Degradation Products

Lipases in flour hydrolyze triglycerides into glycerol and free fatty acids, while phospholipases release free fatty acids from phospholipids [27,28]. In high-temperature environments like baking and frying, fatty acids lose a proton at the double bond to form alkyl radicals, which oxidize to produce peroxyl radicals [29]. These peroxyl radicals abstract hydrogen atoms from other unsaturated fatty acid molecules, forming hydroperoxides and new alkyl radicals, propagating a chain reaction that generates unstable hydroperoxides [30]. These hydroperoxides undergo β-scission to produce aldehydes and ketones, supplying carbonyl sources for the Maillard reaction (Figure 1B) [31,32]. For example, decomposition of oleic acid hydroperoxides produces octanal, heptanal, nonanal, and 2-nonenal. Linolenic acid hydroperoxide decomposition yields hexanal and 2-pentenal, while linoleic acid decomposition produces hexanal, pentenal, and 2-octenal [33]. Lipid oxidation products can interact with Maillard reaction substrates and intermediates to regulate the formation of Maillard volatile products [34]. Aldehydes generated by lipid oxidation, such as 4,5-epoxy-2-alkenals, can react with amino acids to form N-substituted hydroxyl alkyl pyrrole. This intermediate further polymerizes to form brown substances like melanoidins. Concurrently, phospholipid oxidation products can diminish sulfur-containing Maillard reaction compounds while modifying aroma profiles [35].

#### 2.1.3. O-Quinones

In flour product raw materials, polyphenols exist in free, soluble bound, or insoluble bound forms, with the vast majority being bound [36,37]. Grinding of wheat grains destroys cellular structures, releasing polyphenol oxidases (PPOs). PPOs catalyze the oxidation of polyphenols to form o-quinones (Figure 1C) [38,39]. These o-quinones can participate in the Maillard reaction with amino acids and proteins. Whether polyphenols can be oxidized by PPOs to form o-quinones largely depends on their molecular structure. PPOs contain copper ion active centers. Under aerobic conditions, PPOs initiate electron removal from catechol-type polyphenols generating unstable semi-quinone radical intermediates, which rapidly lose additional electrons, forming o-quinones [40]. The main catechol polyphenols in the raw materials of flour products include caffeic acid, chlorogenic acid, quercetin, rutin, protocatechuic acid, catechins, and epicatechins [41,42]. In contrast, although ferulic acid is the most prominent phenolic compound in wheat, it lacks the ortho-dihydroxy group and is not a substrate for polyphenol oxidase. Thus, it cannot form an o-quinone to participate in the Maillard reaction [43].

#### 2.1.4. The Carbonyl Group Provided by Exogenous Excipients

Sucrose and high-fructose corn syrup additions primarily enhance flour product taste through their sweetening properties, while simultaneously influencing the Maillard reaction. Although sucrose, as a non-reducing sugar, cannot initiate the Maillard reaction, it hydrolyzes into glucose and fructose during processing, thereby increasing carbonyl compound availability. In baking goods, high-fructose corn syrup is often used to replace sucrose for economic and technological considerations [45]. The glucose and fructose in high-fructose corn syrup are reducing sugars and can directly increase the substrate of the Maillard reaction. The lactose in the added dairy products is composed of glucose and galactose linked by β-1,4 glycosidic bonds. In solution, it can be converted into an open-chain form, exposing the free aldehyde group (glucose end), which endows it with the typical properties of a reducing sugar and makes it a carbonyl donor in the Maillard reaction [16].

### 2.2. Amino Sources

#### 2.2.1. Amino Acids

##### Natural Free Amino Acids in Raw Materials

The types and contents of free amino acids differ among raw materials for various flour products. Asparagine, aspartic acid, glutamic acid, and alanine are common amino acids in most raw materials. Total free amino acid contents range from 1299 to 2314 mg/kg: rye contains the highest content, followed by oat and buckwheat, with barley and wheat showing comparatively lower levels [18,46]. The free amino acid contents in different raw materials are shown in Table 1.

##### Amino Acids from Protein Hydrolysis

Flour contains various proteins, such as gliadin, glutenin, globulin, and albumin. During processing, the structures of some proteins are disrupted. Endogenous proteases naturally present in flour initially hydrolyze these proteins into small polypeptide fragments under appropriate temperature and humidity conditions [47]. Subsequently, lactic acid bacteria further convert these peptides into amino acids via strain-specific endopeptidases, providing amino groups for the Maillard reaction [48].

#### 2.2.2. Protein Amino Terminus

The α-amino group at the N-terminus of proteins exhibits strong nucleophilicity due to the lone pair of electrons on the nitrogen atom, enabling it to directly participate in the Maillard reaction [49]. Taking glutenin as an example, the N-termini of its high-molecular-weight and low-molecular-weight subunits can bind to carbonyl compounds to initiate the reaction. Additionally, protease hydrolysis during flour product processing generates new polypeptide cleavage sites with reactive N-termini that can participate in the Maillard reaction. Salt is often added to the dough to increase its strength. Salt addition neutralizes gluten protein surface charges and disrupts intermolecular hydrophobic interactions. This promotes extended protein conformations that expose additional amino termini. Consequently, subsequent Maillard reactions are enhanced [50].

#### 2.2.3. The Amino Groups Provided by Exogenous Excipients

Dairy products are often added as ingredients in the production of flour products (bread and biscuits). Dairy products have a relatively high protein content and can serve as an amino donor for the Maillard reaction. The protein in dairy products is mainly casein, whose molecular structure contains multiple free amino groups, especially the ε-amino group of lysine residues. These free amino groups are exposed in the primary structure of proteins and can directly undergo the Maillard reaction with the carbonyl groups of reducing sugars without hydrolysis [51].

## 3. Maillard Reaction Process and Influencing Factors in Flour Product Processing

As an important chemical reaction in food thermal processing, the Maillard reaction process is well-studied. Researchers generally divide it into three stages: the initial, intermediate, and final stages [52]. The reaction pathways at each stage are synergistically regulated by substrate composition and process parameters such as temperature, pH, and moisture content.

### 3.1. Reaction Process

#### 3.1.1. Initial Stage

The carbonyl groups (C=O) of reducing sugars (aldoses or ketoses) undergo a nucleophilic addition reaction with the amino groups (-NH_2_) of amino compounds (e.g., amino acids, peptides, or proteins), forming unstable adducts. Subsequently, the adducts dehydrate to form a Schiff base, and the Schiff base undergoes molecular rearrangement. Aldoses (e.g., glucose) experience Amadori rearrangement to yield Amadori rearrangement products (ARPs), which have the chemical structure of 1-amino-1-deoxy-2-ketoses. Ketoses (e.g., fructose) undergo the Heyns rearrangement to produce Heyns rearrangement products (HRPs), whose chemical structures are 2-amino-2-deoxyaldoses [53,54,55]. Initial reaction products are colorless and odorless but serve as crucial precursors for intermediate stage reactions.

#### 3.1.2. Intermediate Stage

The intermediate stage of the Maillard reaction exhibits pH-dependent pathway divergence. Under acidic conditions (pH ≤ 7), a 1,2-enolization reaction occurs. The rearrangement product first undergoes tautomerization, and then dehydrates to increase the number of double bonds in the molecular structure. Subsequently, water-catalyzed keto-enol tautomerization and water molecule addition eliminate amino acids, followed by dehydration proton transfer and cyclization forming cyclic intermediates. Finally, HMF is formed through intracyclic dehydration (with hexose as the reducing sugar), and furfural is formed when pentose is the reducing sugar. The Maillard intermediate product HMF is closely related to browning [54]. Under alkaline conditions (pH > 7), 2,3-enolization occurs, producing unstable reductones that readily isomerize into dehydroreductones [56]. Under alkaline conditions (pH > 7) and at high temperatures, the rearrangement products generate many active intermediates, such as acetol, methylglyoxal (MGO), and diacetyl groups. These active intermediates and dehydrogen-reducing ketones can undergo Strecker degradation reactions with amino acids to form aldehydes and α-amino ketones. Strecker degradation is associated with browning and the formation of flavor [54,57,58].

#### 3.1.3. Final Stage

Aldehydes and ketones produced in earlier stages condense to form unsaturated carbonyls (aldehydes and enols). These compounds then undergo aldol condensation and polymerization with nitrogen-containing amino compounds, forming complex macromolecular nitrogenous brown polymers or copolymers known as melanoidins [59,60]. Concurrently, highly reactive α-dicarbonyl compounds (e.g., 3-deoxyglucosone (3-DG), glyoxal (GO), and MGO) interact with free amino groups (primarily on lysine and arginine) to form stable AGEs [56].

The Maillard reaction is the most common type of non-enzymatic browning. Although its fundamental mechanism is understood, the specific pathways in the final stage remain incompletely elucidated due to the reaction’s complexity. Quantum chemistry calculations have emerged as a valuable tool for investigating the intrinsic mechanisms of chemical reactions at a microscopic level. These calculations can reveal complex reaction pathways through precise simulations of molecular structure, energy changes, and electron transfer [54]. More quantum chemical studies on Maillard reaction mechanisms are anticipated.

### 3.2. Influencing Factors

#### 3.2.1. Reaction Substrates

(1) Type and content of carbonyl compounds

Reducing sugars are essential substrates for the Maillard reaction. Reaction rates decrease with increasing molecular weight but increase with more side chains. Monosaccharides exhibit stronger glycosylation ability and higher reaction rates than polysaccharides [61]. Due to lower steric hindrance, aldoses with terminal carbonyl groups react more readily with amino acids than ketoses. Among similar flour products, pentoses (xylose and arabinose) react faster than hexoses (glucose and fructose) and disaccharides (maltose), with glucose reacting faster than fructose. Across different flour products, higher reducing sugar content accelerates the Maillard reaction. For instance, the bran in whole wheat products contains more arabinoxylan [62]. Arabinoxylan degradation generates additional reducing sugars, potentially leading to a greater extent of Maillard reaction compared to refined wheat flour products [63].

(2) Type and content of amino compounds

The reactivity of amino compounds is mainly related to the stereospatial structure of the amino group, and the greater the spatial resistance, the weaker the reactivity. Among amino acids, longer carbon chains correlate with lower reaction rates. Amino acids with amino groups at the terminal or ε-position are more prone to the Maillard reaction than those with amino groups at the α-position. Basic amino acids are more susceptible than acidic amino acids. Common amino compounds follow the following reactivity order in the Maillard reaction: amines > amino acids > proteins [56]. Since reducing sugar content is relatively high in flour product raw materials, amino acid content is often the most important or rate-limiting factor affecting the Maillard reaction [19].

#### 3.2.2. Heat Input

Temperature and time are important factors affecting the Maillard reaction. In general, the typical Maillard reaction occurs above 120 °C. Thus, it is prominent in baked and fried flour products, while minimal in steamed and boiled products. However, low-temperature Maillard reactions also occur during storage of boiled flour products, exemplified by the browning of fresh noodles. The reaction rate increases with temperature. A 10 °C increase can accelerate the Maillard reaction rate by 3- to 5-fold [56]. Appropriate heat input enhances surface color uniformity and flavor intensity in flour products. Excessive temperature or prolonged time accumulates melanoidins, causing over-browning, nutrient destruction, and toxic substance formation.

#### 3.2.3. Moisture

Water content critically influences Maillard reaction progression. The optimal occurrence arises at 30–75% moisture content, where reaction rates increase with rising water levels [64]. Moisture affects the Maillard reaction by influencing the mobility and availability of reactants. In the later stage of the baking and frying of flour products, when moisture content is suitable, reactants have sufficient molecular diffusion coefficients without excessive dilution, resulting in relatively fast Maillard reaction rates [56]. High moisture dilutes reactants and slows the reaction [65]. It also hinders the formation of key flavor compounds like pyrazines and thiazoles [66]. In addition, elevated moisture also promotes caramelization, which consumes reducing sugars through continuous dehydration and decomposition reactions, thereby competing with the Maillard reaction for substrates. Conversely, very low moisture limits reactant mobility and reduces intermolecular collision frequency, inhibiting the reaction. Moisture content also influences the enolization pathway of the Maillard reaction. Under low moisture content conditions, 2,3-enolization is more likely to occur. Low moisture favors 2,3-enolization, while increasing moisture shifts the mechanism towards 1,2-enolization [65].

Unlike the total moisture content, water activity (a_w_) is a parameter reflecting the participation of water in chemical reactions. It affects the availability of reactants and the mobility of molecules in the reaction system. Generally speaking, when the water activity is 0.60 to 0.70, the Maillard reaction rate is the highest. Low water activity can lead to reduced reaction rates or incomplete reactions, resulting in undesirable sensory properties such as burnt or bitter tastes of flour foods [67]. High water activity leads to a shift towards the caramelization reaction, generating more caramelization reaction products rather than Maillard reaction products [68].

#### 3.2.4. pH

pH influences the Maillard reaction by affecting the ionization state and reactivity of reactants and intermediates. Within the pH range of 3 to 9, the reaction rate generally increases with pH. In fermented flour products containing organic acids, the acidic environment promotes hydrolysis of N-glycosylamines (Schiff base), reducing the reactants available for the intermediate stage and inhibiting further Maillard progression [56]. In neutral or slightly alkaline flour products, amino acids are more prone to deprotonation, enhancing their nucleophilicity. Additionally, high pH favors sugar molecular rearrangement, promoting nucleophilic addition reactions and increasing the Maillard reaction rate [59,69]. pH also alters the reaction pathway and flavor compound profile. Amadori compounds undergo 1,2-enolization at pH 8 but 2,3-enolization at pH 9.7. Low pH favors furan formation, while high pH promotes pyrazine formation [59].

## 4. Outcomes of the Maillard Reaction in Flour Food Processing

### 4.1. Color and Browning

**Figure 2:**
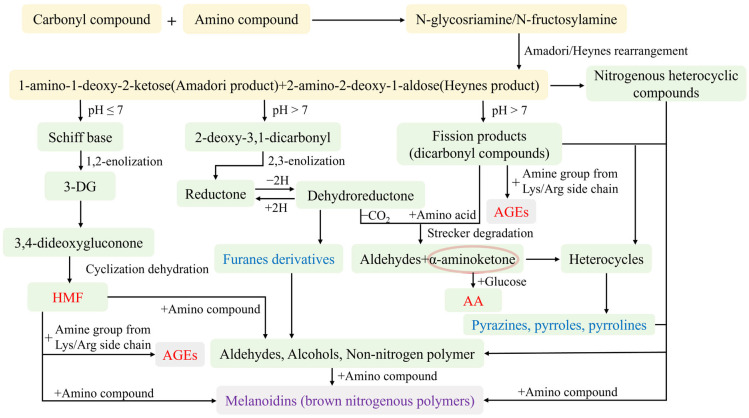
Formation of Maillard browning products, flavor substances, and harmful products [33]. The yellow, green, and gray bottom colors refer to the initial, intermediate, and final stages in the reaction, respectively. The violet, blue, and red fonts represent browning products, flavor substances, and harmful products, respectively.

The Maillard reaction forms colorless glycoamine condensation compounds in the initial stage, such as Amadori and Heyns compounds. In the intermediate stage, reductones are formed. The conjugated structure of reductones lowers the energy required for electron transitions, shifting absorbed light wavelengths into the visible range (e.g., blue-violet light, 380–500 nm) and resulting in reflected complementary yellow light (565–590 nm). Consequently, yellow compounds with high ultraviolet absorption are formed. In the final stage, reductones further polymerize with amino acids to form high-molecular-weight brown melanoidins [70]. The formation of browning products is shown in Figure 2.

The rate and degree of browning are affected by the type of reactants [70]. At the usual baking temperature below 300 °C, sucrose is relatively stable, while cookies formulated with fructose produced more HMF, a key intermediate in browning, than those with glucose [71]. The influence of amino acid type on browning varies. Lysine, glycine, tryptophan, and tyrosine cause the most browning, followed by proline and leucine, with histidine and threonine showing the least [72]. The impact of quinone structure on browning depends on ring composition and substituent differences, which alter quinone reactivity and thus affect the browning degree and rate. Compounds containing only a flavonoid A-ring do not cause browning, while those with only a flavonoid B-ring contribute minimally. The synergistic action of A- and B-rings significantly accelerates browning [43].

The browning process of baked flour products is also dynamically regulated by temperature and water activity. Browning can be detected when the temperature exceeds 105–120 °C and the water activity is 0.4–0.7 [73]. The effect of temperature on browning dynamics was significant. The absorbance at 420 nm (maximum absorbance of the end product) of the aqueous extract of bread baked at 225 °C for 50 min was four times higher than that baked at 220 °C for 60 min [70,74,75]. This indicates that the increase in temperature accelerates the reaction process dramatically [75]. Besides the typical high-temperature Maillard reaction, a low-temperature Maillard reaction occurs in various foods with moderate moisture content that have been stored for a long time [76]. A typical representative is the low-temperature Maillard browning of fresh noodles. Maillard products like furosine and HMF have been detected in whole wheat fresh noodles stored at 25 °C. Furosine serves as an index of the initial stage of the Maillard reaction in food products, while HMF is accepted worldwide as a marker for the intermediate stage of the Maillard reaction. [77]. However, research on such low-temperature browning faces challenges due to difficulty distinguishing it from enzymatic browning. Superheated steam treatment can reduce the activity of PPO in wheat flours, thereby inhibiting enzymatic browning, and thus can be used to study non-enzymatic browning of fresh noodles [78]. Spatial moisture distribution also affects browning. During bread baking, water evaporation forms a crust with low moisture content (5–10%), creating optimal conditions for the Maillard reaction. In contrast, the crumb moisture is relatively high, limiting the reaction and resulting in significantly lower browning compared to the crust [70].

### 4.2. Flavor Substances

Flavor is a key characteristic of Maillard reaction products, with flavor compounds initially forming in the intermediate stage (as shown in Figure 2). Sugars have limited influence on flavor characteristics, while amino acids significantly impact them. Different amino acids produce distinct flavors. For example, lysine imparts fruity, pleasant floral, and toasted bread notes; glycine produces caramel; alanine gives fruity and floral fragrances; tyrosine yields rose fragrance; proline generates floral scents and toasted bread flavor; and valine produces chocolate flavor [59].

Maillard-derived flavor compounds include alcohols, esters, aldehydes, ketones, oxygen-containing heterocyclics (e.g., furfurals, furanones, and pyranones), nitrogen-containing heterocyclics (e.g., pyrazines, pyrroles, pyridine, and pyrrolines), and sulfur-containing heterocyclics (e.g., thiazoles, thiophenes, and thiazolines) [79]. Alcohols contribute alcoholic and rose notes; aldehydes impart fruity and malt flavors; ketones offer creamy and caramel aromas; and pyrazines provide nutty and roasted notes [80,81,82,83]. Furan derivatives contribute sweet and caramel aromas characteristic of bread [84]. Flavor compounds in different flour products are summarized in Table 2.

The Maillard reaction generates diverse flavor compounds that impart unique sensory characteristics to flour products, yet it may also produce undesirable odorous subs tances under high temperatures, prolonged processing, or extended storage conditions, compromising organoleptic quality. For instance, this includes pungent odors such as those thiazole produce at excessively high temperatures, and elevated hexanal concentrations emit rancidity [59]. High concentrations of 2-pentylfuran impart a beany, cardboard-like, and stale oil smell [85].

**Table 2:** Flavor substances in different flour products [2,79,86,87,88,89,90].

Category	Breads	Cookies	Fried Bread Sticks	Steamed Breads
Alcohols	Ethanol Nonanol 1-Propanol 1-Butanol 1-Octen-3-ol 2,3-Butanediol Phenethyl alcohol	n-Octanol 2,3-Butanediol 2-Nonyl alcohol Phenethyl alcohol	1-Octen-3-ol Heptanol Octanol	1-Heptanol 1-Hexanol 1-Pentanol Phenethyl alcohol 1-Octen-3-ol 2-Phenylethanol
Esters	Ethyl formate Ethyl acetate Ethyl valerate γ-Nonalactone	Ethyl nonanoate Ethyl caprylate Ethyl caprate	γ-Nonalactone	Ethyl Lactate Ethyl acetate Hexenyl butyrate γ-Nonalactone
Aldehydes	Hexanal Nonanal Heptanal Benzaldehyde (E)-2-Nonenal (E)-2-Heptenal (E)-2-Octenal 2-Methylbutanal 3-Methylbutanal	2-Methylpropanal 2-Methylbutanal Hexanal Nonanal Benzaldehyde	Hexanal Heptanal Nonanal Decanal (E)-2-Hexenal (E)-2-Octenal (E)-2-Nonenal	Nonanal Hexanal Octanal Benzaldehyde (E,E)-2,4-Decadienal (E)-2-Nonenal
Ketones	2-Pentanone 2-Heptanone 1-Octen-3-one 2,3-Pentanedione 3-Hydroxy-2-butanone 1-Hydroxy-2-propanone 2,3-Butanedione 3-Hydroxy-2-butanone	Methyl-heptenon 2-Heptanone	3-Hydroxy-2-butanone	Octanone Acetophenone Geranylgeranylacetone 2,3-Pentanedione
Oxygen-containing heterocyclic compounds	5-Methylfuranal 2-Furylcarbinol 2-Pentylfuran 2-Methyl furan Furfural	Furfural	2-Pentylfuran 2,5-Dimethyl-4-hydroxy-3(2H)-furanone Furfural	Dihydro-5-pentyl-2(3H)-furanone 2-Pentylfuran
Nitrogen-containing heterocyclic compounds	2-Methylpyrazine 2-Acetyl-1-pyrroline 2,3-Dimethylpyrazine 2,5-Dimethylpyrazine 2,6-Dimethylpyrazine 2,3,5-Trimethylpyrazine	2-Methylpyrazine 2,3-Dimethylpyrazine 2,5-Dimethylpyrazine 2,6-Dimethylpyrazine 2-Acetylpyridine	2-Ethyl-3,5-dimethylpyrazine 3-Ethyl-2,5-dimethylpyrazine 2,3-Dimethylpyrazine	
Sulfur-containing heterocyclic compounds	1,3-Thiazole 2-Acetyl-2-thiazoline		2-Acetylthiazole	

Due to the complexity of food components and the diversity of food processing conditions, model reactions are often used to explore flavor formation pathways. Isotope labeling techniques, particularly the carbon module labeling (CAMOLA) method, are currently employed to investigate the formation pathways of flavor compounds [91]. The reaction of [1-^13^C]-ribose with cysteine suggests that furfural is the intermediate for the formation of 2-furfurylthiol, while 1,4-dideoxypento-2,3-diulose is that for the generation of 2-methyl-3-furanthiol and 3-mercaptopentan-2-one [92]. Similarly, the reaction of cysteine with [^13^C_6_]-glucose demonstrates that 2-acetylthiazole can derive from GO and MGO from glucose reacting with H_2_S and NH_3_ via Strecker degradation of cysteine [93].

In addition to sulfur-containing flavor compounds, researchers have also investigated the formation pathways of nitrogen-containing heterocyclic compounds, especially pyrazine compounds with baking flavor characteristics in cereal foods. Labeling lysine with ^15^N at the α-amino group and ^14^N at the ε-amino group shows both amino groups contribute to pyrazine formation during lysine–glucose reactions. However, The α-amino group reacts more readily with dicarbonyls than the ε-amino group to form pyrazines [94].

### 4.3. Nutritional Changes

During the Maillard reaction, some substances with antioxidant activity are produced, among which the most typical one is melanoidins. Melanoidins are a group of anionic and colored compounds generated during the Maillard reaction. High-molecular-weight melanoidins exhibit superior antioxidant activity compared to low-molecular-weight counterparts, primarily through free radical scavenging such as DPPH and ABTS and metal chelation [95]. The antioxidant activity depends on sugar type, added quantity, and baking parameters. Research demonstrated that ribose-generated Maillard reaction products in bread crusts achieve the strongest antioxidant effects, followed by fructose and sucrose, with maltose and glucose showing minimal activity. Increasing sugar addition from 6 g to 12 g per 100 g flour enhanced the antioxidant activity of the bread crust. In addition, extending the baking time from 24 to 40 min was more effective in enhancing the antioxidant activity than raising the baking temperature from 215 °C to 230 °C [96].

The Maillard reaction reduces the bioavailability of essential amino acids and the digestibility of proteins [97]. Condensation between reducing sugars and amino acids during the reaction destroys the structure of amino acids. Lysine, being the first limiting amino acid in cereals with a highly reactive ε-amino group, exhibits significant bioavailability loss. Browning also involves oxidation and destruction of other essential amino acids and protein cross-linking, damaging protein digestibility and reducing the nutritional quality of baked cereal foods [98]. For example, Tsen et al. [99] reported that baking decreased the protein efficiency ratio (PER) of bread dough from 1.34 to 0.92. Compared to the bread crumb (90% lysine availability), the crust had only 75% availability, demonstrating the Maillard reaction’s negative impact on nutritional value.

### 4.4. Harmful Products

#### 4.4.1. Acrylamide

AA is the most prevalent harmful product in heat-processed (e.g., fried and baked) flour products. AA content varies among products. Bread, cookies, and fried bread sticks contain 6.66–134.8, 26.75–384.5, and 21.34–2095.2 μg/kg, respectively [100,101]. The Commission Regulation (EU) 2017/2158 stipulates the benchmark levels of AA in different foods. The benchmark levels for breakfast cereals, biscuits, and wafers are between 150 μg/kg and 800 μg/kg. That for infant biscuits and bread is 150 μg/kg. There are mainly two formation pathways of AA. Firstly, it is produced by asparagine and reducing sugars through the Maillard reaction at around 120 °C [102,103,104], and reaches its peak at 160–180 °C. Secondly, AA is formed through dehydroxylation reactions between acrolein and acrylic acid produced by lipid degradation and amino acids or ammonia produced during protein pyrolysis [105,106]. AA is neurotoxic, causing nerve damage [12]. Long-term low-dose exposure may adversely affect the nervous system, such as inducing peripheral neuropathy [107]. Furthermore, animal studies indicate AA is also potentially carcinogenic and mutagenic [13].

#### 4.4.2. 5-Hydroxymethylfurfural

The content of HMF in specific cereal foods depends on the processing type. Cookies and breads contain 1.65–82.78 mg/kg and 0.66–18.34 mg/kg HMF, respectively [108]. There are mainly two formation pathways of HMF in the processing of flour products. The first pathway is the Maillard reaction. HMF is formed in the medium stage of the Maillard reaction through Amadori rearrangement (Figure 2). The second is that sugars undergo caramelization reactions under acid catalysis and heat treatment. For instance, sucrose decomposes into glucose and fructose. These sugars then undergo enolization and dehydration to form fructofuranosyl cations. At specific temperatures, these cations convert to HMF [108,109]. HMF exhibits multiple negative effects. In terms of carcinogenicity, it promotes the growth of tumor cells. Regarding genotoxicity, HMF is activated by sulfotransferases in the body, converting into SMF that induces gene mutations and thereby exerts toxicity on the genome. HMF also has mutagenicity, capable of altering the genetic material of organisms. Additionally, a high-dose intake of HMF can irritate the skin, respiratory tract, eyes, and mucous membranes [14]. Its metabolite SMF is toxic to the kidneys [108].

#### 4.4.3. Advanced Glycation End Products

More than 20 kinds of AGEs have been discovered until now. The most important and common ones are CML and CEL [110]. The contents of AGEs in different flour products also varied (Table 3) [100]. AGEs are mainly produced through the following pathways. The Schiff base in the Maillard reaction rearranges into Amadori or Heyns products. These products undergo oxidation, deamination, and dehydration to form α-dicarbonyl compounds (e.g., GO, MGO, and 3-DG), which react with lysine, arginine, or other amino acid residues to yield AGEs (as shown in Figure 2). Additionally, AGE precursors (α-dicarbonyls) can arise from reducing sugar autoxidation, lipid peroxidation, and oxidative cleavage of the Schiff base [111,112]. The accumulation of foodborne AGEs in the human body can be harmful. Research suggests AGEs may induce oxidative stress or damage nerve cells, potentially leading to various diseases [15].

## 5. Mitigation Strategies for Maillard Reaction Harmful Products in Flour Product Processing

**Figure 3:**
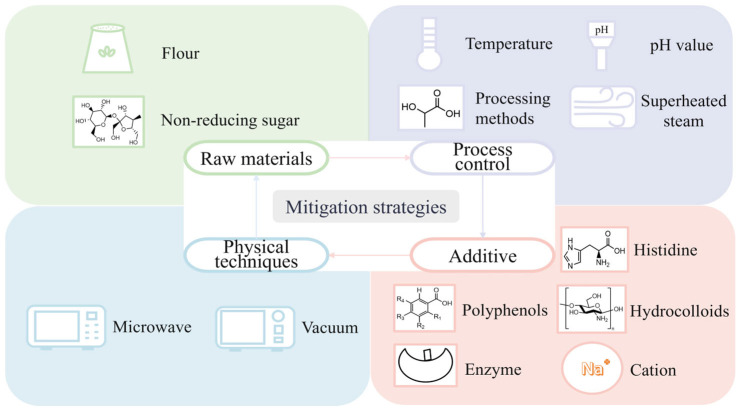
Mitigation strategies for Maillard reaction harmful products in the processing of flour products.

Although existing strategies have made progress in inhibiting harmful products (such as AA and AGEs), synergistic optimization of color and flavor remains inadequate. For instance, polyphenols inhibit harmful products but may mask baking aromas. Similarly, low-temperature processes reduce harmful substances but weaken flavor compound accumulation. Therefore, developing comprehensive, multidimensional strategies is crucial for balancing sensory quality and safety. Existing mitigation methods are discussed below, covering raw material selection and processing, processing control, additive applications, and physical techniques (Figure 3).

### 5.1. Raw Material Selection and Processing

#### 5.1.1. Selection of Suitable Flour

Selecting flours with lower asparagine content is an effective strategy to reduce AA production in flour products. Asparagine is a key precursor for the formation of AA. During high-temperature processing, asparagine undergoes the Maillard reaction with reducing sugars, which is the main method for AA generation [103]. When the asparagine content in flour is low, the amount of asparagine participating in the Maillard reaction decreases, thereby mitigating the formation of AA. Studies showed that hulled oat, durum wheat, and rye flours had relatively high asparagine levels (859.8, 603.2, and 530.3 mg/kg, respectively), resulting in higher AA levels after baking compared to other varieties. Refined bread wheat flour and red corn flour had a lower asparagine content, leading to significantly reduced AA formation [114].

Selecting flours with a low total amino acid content can mitigate the formation of AGEs in flour products. Furthermore, specific amino acids—notably lysine, arginine, and cysteine—distinctly influence the types of AGEs formed. For instance, arginine levels affect CML generation, while cysteine is linked to the production of fluorescent AGEs. Consequently, choosing flour based on its specific amino acid profile offers a targeted strategy to effectively suppress the formation of harmful Maillard reaction products [115].

Bread, noodles, and biscuits typically employ high-, medium-, and low-gluten wheat flours, respectively, to satisfy the distinct gluten strength requirements. Flour with a higher gluten content contains increased protein, including amino termini, which potentiate a stronger Maillard reaction. In addition, dough-added salts and alkalis further regulate these reactions by modifying the gluten protein structure. Among them, neutral salts (e.g., NaCl) cause the gluten to expand and form a strip-like structure, exposing more amino terminals [50]. However, alkaline salts (e.g., Na_2_CO_3_), although they promote the cross-linking and aggregation of gluten proteins, can prevent the protonation of amino groups and enhance reactivity, thereby influencing the direction and intensity of the Maillard reaction [116].

#### 5.1.2. Optimization of Reducing Sugar

The use of non-reducing sugars to replace reducing sugars is an effective way to inhibit the formation of harmful Maillard products. Taking invert syrup as an example, it is rich in reducing sugars such as glucose and fructose, so it is the main precursor for AA formation. Studies showed a positive correlation between invert syrup addition and AA levels [117]. As a non-reducing sugar, sucrose needs to be hydrolyzed into glucose/fructose (reducing sugar) before participating in the Maillard initial reaction. Therefore, partially replacing reducing sugars (e.g., invert syrup) with non-reducing sugars (e.g., sucrose) can delay the occurrence of the Maillard reaction and reduce the formation of AA in flour products.

### 5.2. Processing Control

#### 5.2.1. Optimization of Processing Parameters

The regulation of temperature and time can effectively balance the production of harmful products and flavor substances in the Maillard reaction. Higher baking temperatures (205–230 °C) promote CML formation, while lower temperatures (155–205 °C) favor AA generation during the baking of cookies. A balanced approach (e.g., 180 °C/16 min) mitigated risks but did not fully eliminate harmful compounds [118]. Other studies have shown that low-temperature and long-time fermentation can promote the generation of flavor substances and increase the formation of various key volatile flavor substances in bread, such as 3-methyl-1-butanol, 3-methylbutanal, and 2-methyl-1-propanol [119].

Adjusting the pH value of the system is another key strategy to inhibit harmful products. AA formation is pH-dependent, with lower pH reducing its levels. The addition of tartaric and lactic acid to bread and cookies resulted in a linear decrease in AA levels, while adding citric acid in semi-sweet cookie dough reduced AA by 20–30% [120].

#### 5.2.2. Improvement of Processing Methods

Superheated steam treatment is conducive to the Maillard reaction and relevant formation of surface color of flour products. Rapid steam condensation elevates the surface temperature, altering internal moisture migration and chemical reactions to accelerate browning. Studies have shown that at 200 °C, the L* value of bread under superheated steam conditions decreased from about 85 to 35 in 1800 s, while the decrease was relatively slow in hot air baking. Therefore, the superheated steam method can be used to bake bread [121].

The method of dough preparation has a significant impact on the formation of AA in rye bread. When preparing dough by the direct method, the pH of the dough was reduced by acidifying the dough with the addition of lactic acid, which inhibited the formation of the AA precursor Schiff base. It was shown that the AA content in rye bread produced by the direct method (acidifying the dough with lactic acid) was 3.5 times lower than that in rye bread produced by the indirect method (fermenting the dough with a leavening agent) [122]. Therefore, the direct method can be used to prepare dough to reduce the AA content in rye bread.

Optimizing pasta drying processes effectively reduced harmful Maillard reaction byproduct generation. During the initial drying stages when the moisture content exceeds 15% and water activity remains high, implementing rapid high-temperature treatment inhibited intermediate-stage HMF formation. This required promptly elevating the temperature to 81 °C within 25 min and maintaining it for 2.5 h. Concurrent control of heating rates and thermal gradients reduced water activity and diminished reducing sugar accessibility. These integrated measures avoided the destruction of the gluten network structure while limiting reducing sugar–amino acid interactions, collectively reducing Maillard reaction harmful compound generation without compromising cooked pasta quality [53].

### 5.3. Additive Applications

#### 5.3.1. Polyphenols

Polyphenols can regulate the Maillard reaction and inhibit harmful products in flour products [123,124]. Inhibitors include phenolic acids and flavonoids and the regulatory mechanisms mainly include trapping α-dicarbonyl compounds, scavenging free radicals, chelating with metal ions, and shielding the amino structure in proteins [110,125,126,127].

Flavonoids with 3-OH and 5-OH groups (such as catechins and quercetin) can trap α-dicarcarbonyl compounds, thereby reducing the content of CML in bread [128,129]. Quercetin can also inhibit the formation of HMF in buckwheat bread by combining with HMF and its precursors through intermolecular dehydration to form adducts [130].

Hydroxycinnamic acid derivatives (such as caffeic acid and ferulic acid) combine with free radicals to form stable molecules through the hydrogen supply of their vicinal hydroxyl groups. The free radical-mediated chain reaction is terminated, inhibiting the formation of AGEs in the bread system [128].

The functional groups, such as hydroxyl and carboxyl groups, in polyphenols may chelate with metal ions. The lone pairs of electrons of hydroxyl and carboxyl oxygen atoms can form coordination bonds with metal ions. This intramolecular encapsulation reduces metal ion activity and inhibits their catalysis of Maillard reactions and oxidation processes [128].

Under alkaline conditions, polyphenol-derived quinones cross-link nucleophilic protein groups (e.g., –NH_2_ and –SH), limiting Maillard substrate availability [128,131]. For example, ferulic acid quinones bind wheat gluten –SH/–NH_2_ groups, blocking carbonyl attachment and the subsequent Maillard reaction [132].

Although polyphenols can inhibit the generation of harmful Maillard products, the bitterness and astringency they introduce can interfere with the Maillard flavor system. Studies have shown that after adding 2.0% of caffeic acid, gallic acid, ferulic acid, catechin, and quercetin to the dough, the Maillard volatile substances decreased by 75.9%, 74.3%, 65.6%, 62.4%, and 59.3%, respectively [133]. Moreover, when the Maillard reaction was insufficient due to the intervention of polyphenols, the color of the baked cereal foods was poor. On the other hand, the browning of polyphenols themselves at high temperatures could partially compensate for the color loss due to the inhibition of the Maillard reaction. This flavor attenuation effect and color compensation mechanism reveal the complex role of polyphenols in food systems, so the selection of polyphenols for regulation of the Maillard reaction in flour food processing needs to be weighed between the inhibition effect and sensory impact.

#### 5.3.2. Hydrocolloids

Hydrocolloids usually have excellent hydrophilicity and thickening properties. They can form a physical barrier layer around the reactant molecules to inhibit the formation of harmful products from the Maillard reaction [134]. Gum Arabic can form a tight coat on the surface of biscuits, inhibiting water evaporation and altering the reaction environment to reduce the generation of harmful products such as AA and HMF [135].

Hydrocolloids inhibit harmful Maillard reaction products by lowering the pH. Their acidic groups (e.g., carboxyl groups) release hydrogen ions to reduce the pH value. This low-pH environment hinders electrophilic addition reactions. For example, the galacturonic acid residues of pectin can lower the pH of dough and inhibit the formation of AA. Experiments showed that adding 5% pectin significantly reduced dough pH and decreased biscuit AA content by 67%, without compromising taste or color [136]. Similarly, dicarbonyl compounds (such as GO and MGO) are more likely to form in high-pH environment. Adding κ-carrageenan can lower the pH value of the dough, inhibiting the formation of dicarbonyl compounds in the cake, and thereby reducing the generation of AGEs [137].

#### 5.3.3. Enzymes

Enzymes exhibit bidirectional regulatory characteristics on the Maillard reaction. Some enzymes can promote the Maillard reaction. Proteases hydrolyze proteins to produce free amino acids and small molecule peptides [138]. The increased content of free amino groups accelerates the Maillard reaction. Similarly, Amylase can hydrolyze starch into reducing sugars such as glucose and maltose [139], providing more carbonyl groups and promoting the Maillard reaction. Moreover, lipase can catalyze the hydrolysis of fats to fatty acids and glycerol. In baked goods, fatty acids in oils and fats can generate aldehydes and ketones after being oxidized by lipase. The content of carbonyl groups is increased to enhance the flavor and attractive color produced by the Maillard reaction [66].

Some enzymes can also inhibit the reaction through decomposing the substrate. For example, L-asparaginase catalyzes the hydrolysis of L-asparagine to form L-aspartic acid and ammonia. By reducing the amount of asparagine, L-asparaginase significantly reduced the production of AA [140]. It has been shown that AA levels in sweet bread and cookies with L-asparaginase added decreased by 81% and 84%, respectively [141].

#### 5.3.4. Cations

Na^+^ and K^+^ can increase furfural substances to promote browning. This browning can improve the color of food, endowing flour products such as biscuits with unique colors, and enhancing their appearance quality. Different cations have different effects on browning, and the appropriate cations can be selected according to the demand. When making cookies, if a browning effect similar to that of traditional sodium-containing products is desired and a reduction in sodium intake is desired, KCl can be used to replace NaCl to maintain the browning effect while achieving sodium reduction [142].

Cations inhibit AA production by causing the reaction pathway to proceed in the direction of glucose dehydration to produce HMF and furfural. For example, Na^+^, K^+^, Mg^2+^, and Ca^2+^ can promote the conversion of glucose to fructose in wheat flour dough. This transformation can reduce the formation of pyrazine and Strecker aldehydes [143]. The effectiveness of the above-mentioned cation-mediated competitive mechanisms of reaction pathways has been experimentally verified: the addition of NaCl at 180 °C and 190 °C reduced the AA content in cookies [144].

#### 5.3.5. Amino Acids

Adding specific amino acids can inhibit browning and harmful product formation in the Maillard reaction. L-cysteine can alter the degradation pathway of ARPs to generate stable cyclic 2-threityl-thiazolidine-4-carboxylic acid (TTCA). This structural analogue has a low degree of degradation, which can reduce the formation of short-chain α-dicarbonyl compounds such as GO, thereby reducing the accumulation of fluorescent compounds and inhibiting excessive browning [145]. Histidine inhibits the formations of HMF and AA by competing with asparagine for glucose. Research found that adding 2% histidine inhibited HMF and AA in cookies by 90% and 65%, respectively, without significantly affecting sensory quality [146].

### 5.4. Physical Processing Techniques

#### 5.4.1. Microwave Processing

A microwave is an electromagnetic wave with a frequency range of 300 MHz to 300 GHz [147]. Microwaves can generate heat energy inside the food without any medium as a heat transfer carrier. Therefore, they can rapidly increase the temperature of flour products in a short time and inhibit the excessive progress of the Maillard reaction [148]. Compared with conventional frying, microwave frying reduced the production of AA by 37–83% [149]. The regulation of the Maillard reaction by microwave depends on the processing parameters and the characteristics of the processed products. Excessive microwave treatment time and high power can lead to higher levels of AA and HMF in the biscuits [150]. Therefore, it is necessary to use appropriate microwave parameters when processing baked foods to inhibit the formation of harmful Maillard products.

#### 5.4.2. Vacuum Treatment

Vacuum treatment can reduce the oxygen content. Some oxidation steps in the Maillard reaction rely on the participation of oxygen. The reduction of oxygen inhibits the oxidation reaction, thereby slowing down the Maillard reaction process. Vacuum treatment also accelerates water evaporation and adjusts the moisture content and distribution inside the flour products, causing them to deviate from the optimal conditions for the Maillard reaction, thus inhibiting the reaction [150]. Palazoglu et al. [151] found that after 7.5 min of traditional baking, reducing the pressure to 60 kPa and then baking for 2.5 min, the AA content of the cookies decreased by 45% compared with baking under normal pressure for 11 min. Therefore, the content of AA can be reduced through vacuum combined baking. The study of Yıldız et al. [152] also showed that the AA content of cookies prepared by vacuum combined baking was 30% lower than that of traditional baked samples. Table 4 summarizes the mitigation methods of the Maillard reaction harmful products in the processing of flour products.

## 6. Conclusions

The Maillard reaction plays a pivotal role in flour product processing, significantly influencing color, flavor, nutrition, and safety. Existing research has elucidated key substrate sources, reaction pathways, and critical factors such as temperature, moisture, and pH. Current mitigation strategies effectively reduce harmful products like acrylamide and advanced glycation end products through raw material selection, process optimization, and additive applications. However, these approaches often focus narrowly on hazard reduction, leaving gaps in understanding the molecular mechanisms of reaction pathways and the synergistic optimization of sensory attributes.

Future research should prioritize four key areas. First, sophisticated analytical methods must be employed to decode the intricate molecular transformations occurring during the terminal reaction phases. Second, comprehensive multi-parameter predictive models need to be established to anticipate and manage reaction behaviors across diverse processing environments. Third, particular attention should be given to elucidating low-temperature Maillard reaction kinetics during product storage, including activation energy determination and moisture–temperature dependency mapping. Fourth, novel integrated regulation approaches should be engineered to concurrently optimize favorable sensory characteristics and reduce potentially harmful compounds. Successful implementation of these research initiatives will facilitate the creation of advanced flour-based products that achieve an optimal equilibrium between exceptional sensory attributes and improved food safety profiles.

## Figures and Tables

**Table 1:** The contents of reducing sugars and free amino acids in different raw materials for flour products.

Type	Barley	Rye	Buckwheat	Oat	Wheat	References
Maltose (g/kg)	10.5 ± 1.7	10.6 ± 0.1	11.6 ± 0.9	4.8 ± 0.3	8.5 ± 0.1	[18]
Glucose (g/kg)	4.9 ± 1.2	4.7 ± 0.1	2.2 ± 0.2	1.7 ± 0.1	1.5 ± 0.1	[18]
Fructose (g/kg)	3.7 ± 0.3	2.7 ± 0.1	0.7 ± 0.1	4.5 ± 0.1	1.3 ± 0.1	[18]
Total free amino acid (mg/kg)	1704 ± 130	2314 ± 59	1960 ± 57	1994 ± 69	1299 ± 33	[18]
Asparagine (mg/kg)	309 ± 16	829 ± 24	113 ± 6	672 ± 22	292 ± 5	[18]
Lysine (mg/kg)	28.6 ± 1.7	16.3 ± 2.7	—	26.5 ± 2.9	18.3 ± 2.5	[19]

Note: — indicates that no data has been reported.

**Table 3:** Contents of harmful products in different flour products.

Type	AA (μg/kg)	HMF (mg/kg)	CML (mg/kg)	CEL (mg/kg)
Breads	6.66–134.8 [100,113]	0.66–18.34 [108]	4.5–617.86 [111]	2.1–71.49 [111]
Cookies	26.75–384.5 [100,113]	1.65–82.78 [108]	0.86–117.53 [111]	3.59–50.79 [111]
Instant noodles	12.91 [100]	—	4.61 [100]	3.39 [100]
Fried bread sticks	21.34–2095.2 [100,101]	—	4.48 [100]	1.99 [100]

Note: — indicates that no data has been reported. AA, acrylamide; HMF, 5-hydroxymethylfurfural; CML, Nε-carboxymethyl-lysine; CEL, Nε-carboxyethyl-lysine.

**Table 4:** Mitigation methods for the Maillard reaction harmful products in flour product processing.

Mitigation Strategies	Types of Flour Products	Mitigation Mechanism	Mitigation Results	References
Selection of raw material				
Using flours with a low content of asparagine	Baking products	Reducing the content of precursor substances of AA	The AA contents of baked products made from refined wheat flour and red corn flour with low asparagine content are lower than those of baked products processed from hulled oat and rye flour.	[114]
Replacing reducing sugar with non-reducing sugar	Cookies	Reducing the content of carbonyl groups	Replacing part of the reducing sugar (invert syrup) with a non-reducing sugar (sucrose) reduces the production of AA.	[117]
Processing control				
Optimization of heat input	Cookies	When the temperature drops, the reaction rate decreases	Reducing the temperature within a short baking time inhibits the generation of CML.	[118]
Adjustment of pH	Cookies	A decrease in pH inhibits the formation of AA	Adding citric acid to the dough of semi-sweet cookies reduced the AA content by 20–30%.	[120]
Optimization of the drying process	Pasta	Reducing the degree of the Maillard reaction	High-temperature rapid treatment reduced the formation of HMF.	[153]
Additive applications				
Adding catechin	Butter biscuit	Trapping α-dicarbonyl compounds, scavenging free radicals, and shielding the amino group in proteins	The contents of free CML and CEL in butter biscuits with 0.3–5% catechins decreased by 31.89–84.19%, and the content of protein-bound CEL decreased by 15.32–30.64%.	[154]
Adding naringenin	Bread	Scavenging free radicals	The contents of CML and total fluorescent AGEs in the bread with 0.25–1% naringenin decreased by 9.67–54.27% and 11.79–35.19%, respectively.	[155]
Adding caffeic acid	Bread	Shielding the amino structure in proteins	The contents of CML in the bread crust and bread crumb with 0.1% caffeic acid decreased by 80% and 50%, respectively.	[128]
Adding quercetin	Bread	Forming adducts with HMF and its precursors	The HMF content in bread with 0.19% quercetin decreased by 86.0%.	[130]
Adding sodium alginate	Cookies	Mitigating the formation of intermediate products for the generation of AA	The AA content in biscuits with 1% sodium alginate decreased by 28%.	[156]
Adding chitosan	Cake	Inhibiting protein oxidation and capturing GO and MGO	The content of AGEs in the cake with 0.5% chitosan decreased by 30.31–61.22%.	[157]
Adding pectin	Cookies	Lowering the pH value	The AA content in biscuits with 5% pectin decreased by 67%.	[136]
Adding gum Arabic	Cookies	Forming a tight adhesive layer to inhibit water evaporation	In the biscuits with 0.28% gum Arabic, AA and HMF decreased by 58.6% and 74%, respectively.	[135]
Adding L-asparaginase	Bread Cookies	Removing asparagine	The AA contents in sweet bread and biscuits with L-asparaginase decreased by 81% and 84%, respectively.	[141]
Adding Na^+^	Cookies	Inhibiting the formation of the Schiff base between reducing sugar and asparagine	The AA contents in biscuits with 0.65% NaCl at 180 °C and 190 °C decreased by 24% and 16.5%, respectively.	[144]
Adding Ca^2+^	Wheat product	Lowering the pH value	The AA content in wheat products with 0.44% CaCl_2_ decreased by 36%.	[158]
Adding histidine	Cookies	Competing with asparagine for glucose	The inhibition rates of AA and HMF in biscuits with 2% histidine were 65% and 90%, respectively.	[146]
Physical techniques				
Microwave treatment	Fried product	Increasing temperature quickly to prevent excessive reaction	The AA content in microwave frying products were 37–83% lower than those in traditional frying ones.	[149]
Vacuum treatment	Cookies	Inhibiting oxidation reaction and accelerating water evaporation	The AA content in the biscuits prepared by vacuum combined baking was 30% lower than that in the samples baked by traditional baking.	[152]

## Data Availability

No new data were created or analyzed in this study. Data sharing is not applicable to this article.

## Abbreviations

The following abbreviations are used in this manuscript:

AA	Acrylamide
HMF	5-Hydroxymethylfurfural
AGEs	Advanced glycation end products
SMF	Sulfoxymethylfurfural
CML	Nε-carboxymethyl-lysine
CEL	Nε-carboxyethyl-lysine
PPOs	Polyphenol oxidases
ARPs	Amadori rearrangement products
HRPs	Heyns rearrangement products
MGO	Methylglyoxal
3-DG	3-Deoxyglucosone
GO	Glyoxal
